# Facile solution growth of vertically aligned ZnO nanorods sensitized with aqueous CdS and CdSe quantum dots for photovoltaic applications

**DOI:** 10.1186/1556-276X-6-340

**Published:** 2011-04-14

**Authors:** Chunyan Luan, Aleksandar Vaneski, Andrei S Susha, Xueqing Xu, Hong-En Wang, Xue Chen, Jun Xu, Wenjun Zhang, Chun-Sing Lee, Andrey L Rogach, Juan Antonio Zapien

**Affiliations:** 1 Department of Physics and Materials Science, City University of Hong Kong, Kowloon, Hong Kong SAR; 2 Center of Super-Diamond and Advanced Films (COSDAF), City University of Hong Kong, Kowloon, Hong Kong SAR; 3 Centre for Functional Photonics (CFP), City University of Hong Kong, Kowloon, Hong Kong SAR

## Abstract

Vertically aligned single crystalline ZnO nanorod arrays, approximately 3 μm in length and 50-450 nm in diameter are grown by a simple solution approach on a Zn foil substrate. CdS and CdSe colloidal quantum dots are assembled onto ZnO nanorods array using water-soluble nanocrystals capped as-synthesized with a short-chain bifuncional linker thioglycolic acid. The solar cells co-sensitized with both CdS and CdSe quantum dots demonstrate superior efficiency compared with the cells using only one type of quantum dots. A thin Al_2_O_3 _layer deposited prior to quantum dot anchoring successfully acts as a barrier inhibiting electron recombination at the Zn/ZnO/electrolyte interface, resulting in power conversion efficiency of approximately 1% with an improved fill factor of 0.55. The *in situ *growth of ZnO nanorod arrays in a solution containing CdSe quantum dots provides better contact between two materials resulting in enhanced open circuit voltage.

## Introduction

As an n-type semiconductor with a direct and wide bandgap of 3.3 eV, ZnO is an attractive material for a variety of applications ranging from ultraviolet lasers [1] and sensors [2] to field-emission devices [3]. In recent years, vertically aligned one-dimensional ZnO nanostructures have gained great interest for dye-synthesized solar cells [4,5], as a promising alternative to mesoporous TiO_2 _films [6]. Both ZnO and TiO_2 _have similar bandgaps, while the higher electron mobility and direct electrical pathways provided by vertically aligned ZnO nanorods/nanowires are favorable for electronic transport [4]. Low cost and large-scale chemical solution-based techniques have been developed to synthesize anisotropic single crystalline ZnO nanostructures on a variety of substrates [5]. Despite the expected advantages, the use of ZnO nanostructures in combination with dyes has been hampered due to their instability in acidic dyes leading to the formation of Zn^2+^/dye agglomerates, an insulating layer blocking the electron injection efficiency from the dye molecules to ZnO [4]. On the other hand, semiconductor nanocrystal quantum dots (QDs) [7] have been considered as promising photosensitizers for TiO_2 _and ZnO-based quantum dot sensitized solar cells (QDSCs) [8] due to their intrinsic attractive properties: bandgap tunable both by the choice of material and by the size offering the possibility to match the solar spectrum, and to align energy levels both in respect to the conduction level of the electron-conducting nanostructure and to redox potential of the electrolyte, and high extinction coefficients [8-12]. Photosensitization of ZnO nanowires/nanorods with CdSe QDs has been reported, with relatively low photocurrents for a photoelectrochemical cell with a liquid triiodide/iodide (I_3_^-^/I^-^) electrolyte due to the low QD coverage resulting in power conversion efficiencies in the range of 0.4-0.6% [13,14]. Those works, however, relied on the use of QDs originally synthesized in organic solvents and thus capped with long-chain organic molecules which had to be post-preparatively exchanged for bifunctional short-chain ligands or thioglycolic acid (TGA) serving as molecular linkers [15] to the oxide surface. Recently, Chen *et al*. reported an improved QDSC by direct loading of mercaptopropionic acid-capped CdSe QDs on TiO_2 _substrates from aqueous solution with a power conversion efficiency of 1.19% [16]. Multilayers of TGA-capped CdTe QDs have been deposited on ZnO nanorods in combination with positively charge polyelectrolyte to improve the light-harvesting ability [17].

In this paper, we demonstrate an efficient coverage of ZnO nanorod arrays (NRAs) grown on a Zn foil substrate by a simple solution approach with CdS or CdSe QDs using water-soluble nanocrystals capped as-synthesized with a short-chain bifuncional TGA linker. We show that the simultaneous use of CdS and CdSe QDs has an advantage of synergetic effect in the light harvest resulting in higher performance for co-sensitized structure compared with the solar cells using only one type of QDs. Furthermore, we demonstrate the modification of the QDSCs by depositing a thin (2 nm) Al_2_O_3 _layer before QDs anchoring to avoid spurious charge transfer at the interface between the electrolyte and Zn metal. Power conversion efficiencies of approximately 1% were obtained using ZnO/Al_2_O_3_/CdSe electrode with an improved fill factor (FF) of 0.55. Besides, *in situ *fabrication of the ZnO NRAs in a solution containing the CdSe QDs results in the enhanced open circuit voltage (*V*_OC_) of approximately 0.72 V.

## Experiment

### Preparation of CdS and CdSe QDs

The CdS and CdSe QDs capped by a short-chain ligand TGA have been synthesized in water as previously reported [18]. In the alkaline solution, carboxylic groups of TGA are deprotonated, serving as anchor points and facilitating the binding of QDs to the oxide surface [15]. Size of the QDs varied between 2 and 2.5 nm.

### Preparation of ZnO NRAs

Zinc foil (99.9%) was ultrasonically washed three times in absolute ethanol, placed in a sealed glass bottle containing 20 ml of de-ionized water, kept at 50°C for 24 h, washed several times with distilled water and ethanol, and finally dried in air. The procedure is a simplified version of a previously reported technique based on the fact that water has the ability to oxidize Zn, in the presence of oxygen, to form ZnO nanorods [19].

For comparison, we have also grown ZnO NRAs using the same procedure as above but adding CdSe QDs to the fabrication solution (15 μl CdSe QDs solution with particle concentration of approximately 10^-4 ^M). The preparation time in this case was 48 h to compensate for a slower growth rate.

### Growth of Al_2_O_3 _for core-shell NRAs

Trimethylaluminum and distilled water, with nitrogen as a carrier gas, were used as precursor and oxidant, respectively, to deposit Al_2_O_3 _by atomic layer deposition (ALD). The deposition temperature was 150°C and the expected growth rate was 0.91 Å/cycle; a total of 15 cycles were carried out to deposit an ultrathin Al_2_O_3 _layer on the surface of selected ZnO NRAs prior to the decoration with QDs.

### Sensitization of ZnO NRAs with QDs

Substrates with vertically aligned ZnO NRAs were immersed in aqueous colloidal solutions of CdS or CdSe QDs (pH 9.5, particle concentration of approximately 10^-5 ^M) for 4 h at room temperature, and subsequently dried at 90°C for several minutes. For co-sensitized ZnO NRAs, the substrates were firstly immersed in aqueous colloidal CdS QDs solutions for 2 h and then in aqueous colloidal CdSe QDs solutions for another 2 h, resulting in the preferential adsorption of CdSe QDs on top of CdS layer. The white color of ZnO covered substrates changes to light yellow or orange after adsorption of CdS or CdSe QDs, respectively.

### Fabrication of photoelectrochemical cells

The photoelectrochemical cells were fabricated as follows. A thin island-like Pt layer has been deposited by dropping 0.8 mM H_2_PtCl_6 _solution on an FTO-covered glass and subsequent annealing at 400°C for 30 min, and used as a photocathode assembled into a cell device face-to-face with ZnO/QD photoanode. The two electrodes were separated by 60 μm spacer and bonded together using compression metal clips. The cell was infiltrated with a liquid I_3_^-^/I^- ^electrolyte containing 0.1 M LiI, 50 mM I_2 _and 0.6 M 1,2-dimethyl-3-propylimidazolium iodide dissolved in acetonitrile, sealed, and characterized immediately owing to the low stability of CdS and CdSe QDs in I_3_^-^/I^- ^electrolyte [20]. The effective electrode area was between 0.2 and 0.5 cm^2^.

### Structural, optical, and electrical characterization

ZnO nanorods were characterized by X-ray diffraction (XRD) spectra recorded with a Siemens D500 diffractometer at 40 kV/30 mA, scanning electron microscopy (SEM; Philips XL 30 FEG), and transmission electron microscope (TEM; a Philips CM20). High-resolution transmission electron microscope (HRTEM) images and fast Fourier transform (FFT) pattern were obtained with a Philips CM200 FEG TEM operated at 200 kV. UV-vis spectra were obtained from diffuse reflectance measurements using an integrating sphere on a LAMBDA 750 UV-vis spectrophotometer. The reflectance spectrum of Zn substrate was used as reference. The current density-voltage (*J-V*) characteristics were recorded with a Ketheley 2400 SourceMeter. The assembled cells were illuminated using a solar simulator at AM 1.5 G, where the light intensity was adjusted with a NREL-calibrated Si solar cell with a KG-5 filter to 1 sun intensity (100 mWcm^-2^).

## Results and discussion

The most widely used fabrication method to obtain vertically aligned ZnO nanostructures is the hydrothermal method [5]. ZnO nanowires on F-doped SnO_2 _(FTO) or In-doped SnO_2 _(ITO) substrates are typically prepared by a two-step approach involving the coating of a substrate with ZnO seed nanoparticles which serve as nucleation sites for the formation of nanowires under hydrothermal treatment [21,22]. Thus, the fabricated ZnO-covered transparent conducting electrode serves as photoanode, through which the cell is illuminated [4]. One of the disadvantages is that the resistance of FTO or ITO glass becomes larger after growing ZnO, which can be detrimental for electronic transport [23]. One-step methods to grow ZnO nanostructures on metal substrates such as Zn foil have been also reported [24,25], given advantage of an easier fabrication (no seeds employed) and lower resistance of substrates.

**Figure 1:**
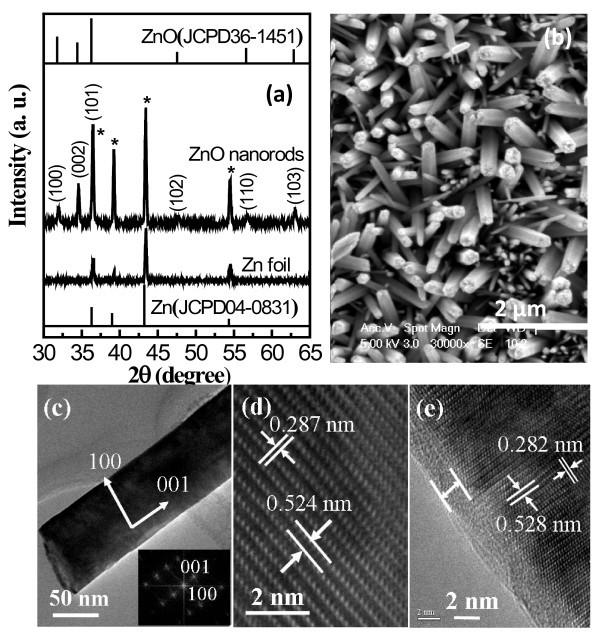
**Structural characterisation of ZnO NRAs**. (a) XRD spectra of ZnO nanorods on Zn substrate and of the bare Zn substrate; (b) top-view SEM image of vertically aligned ZnO nanorods; (c) TEM image of a ZnO nanorod; the inset shows the corresponding FFT pattern that identifies the growth direction as the (001) axis; (d) HRTEM image of a ZnO nanorod showing the lattice spacing and confirming the growth direction; and (e) HRTEM image of a ZnO nanorod coated with a approximately 2 nm Al_2_O_3 _film prepared by ALD.

We have modified a previously reported method of growing large-scale, vertically aligned ZnO NRAs on Zn substrates by a one-step solution-based approach [25]. In the simplified method, this process is improved by employing pure water without the use of additives like the previously used ammonia or hydrogen peroxide. Figure 1a shows the XRD pattern of ZnO NRAs grown on Zn foil. All diffraction peaks can be indexed to hexagonal wurtzite ZnO phase (JCPDS card No. 36-1451) except those marked with * which originate from the zinc substrate (JCPDS card No. 04-0831). Morphological characterization by SEM, Figure 1b, indicates the formation of arrays of ZnO NRs with a preferential growth direction nearly perpendicular to the Zn substrate. The nanorods are uniform in length (~3 μm) and possess a characteristic hexagonal cross-section with diameter in the range of approximately 50 to 450 nm. Higher magnification of a single ZnO nanorod by TEM is shown in Figure 1c; the corresponding FFT pattern indicates that the hexagonal ZnO nanorod grows along the [001] direction. The growth direction is further confirmed by high-resolution TEM image, shown in Figure 1d, which exhibits well-resolved fringes in directions parallel and perpendicular to the nanorod axis, further confirming that the ZnO nanorod is single crystalline. The lattice fringe spacing are 0.524 and 0.287 nm, which agree well with the interplanar spacing of the (001) and (100) planes of hexagonal (wurtzite) ZnO crystals. Selected ZnO NRAs were further treated with an Al_2_O_3 _coating prepared by 15 cycles of an ALD process. Figure 1e shows conformal Al_2_O_3 _deposition with average film thickness approximately 2 nm.

**Figure 2:**
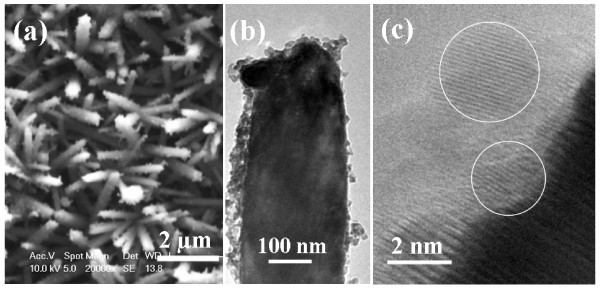
**Structural characterization of QD-decorated ZnO NRAs**. (a) Top-view SEM image of ZnO nanorods decorated with CdSe QDs; (b) TEM of a ZnO nanorod coated with CdSe QDs; (c) HRTEM image of CdSe QDs on a ZnO nanorod, indicated by white circles.

Decoration of the ZnO NRAs with QDs results in significant increase in their surface roughness as presented for the case of CdSe QDs in the SEM image of Figure 2a; furthermore, it is obvious that coverage of the ZnO NRAs has been achieved over large areas. Closer inspection, by TEM of a single ZnO NR in Figure 2b, demonstrates almost complete surface coverage by the CdSe QDs. Figure 2c shows a representative HRTEM image taken at the nanorod's edge and provides further evidence that single crystalline QDs with 2-2.5 nm diameter are directly and tightly attached to the ZnO surface.

**Figure 3:**
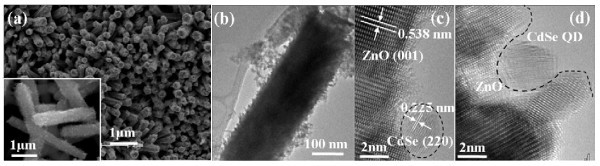
**Structural characterization of ZnO NRAs grown *in-situ *in CdSe QD solution**. (a) SEM image (inset: higher magnification SEM of several nanorods detached from the Zn substrate); (b) TEM image of a ZnO nanorod from sample in (a); (c, d) HRTEM images of a ZnO nanorod surface with incorporated CdSe QDs.

We have also grown ZnO NRAs *in situ *in a water bath containing CdSe QDs. The SEM image in Figure 3a shows that uniform NRAs with orientation which is even closer to the substrate's normal as compared to the pristine ZnO NRAs in Figure 1b. A higher magnification SEM of several NRs detached from the substrate, inset in Figure 3a, shows that the CdSe QDs are incorporated into the ZnO surface resulting in a larger surface roughness. The surface coverage of ZnO with QDs is also confirmed by TEM as shown in Figure 3b. As expected, the incorporation of QDs during ZnO NR growth results in more intimate contact between the ZnO NRs' surface and the QDs as observed in the HRTEM images of Figure 3c,d.

**Figure 4:**
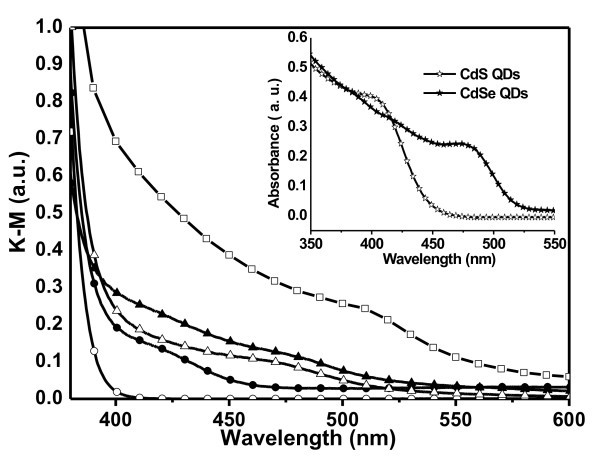
**Kubelka-Munk diffuse reflectance absorption spectra of different samples**. ZnO nanorods (open circle), ZnO nanorods decorated with CdS QDs (filled circle), ZnO nanorods decorated with CdSe QDs (open triangle), ZnO nanorods decorated with both CdS and CdSe QDs (filled triangle), and ZnO nanorods grown in an aqueous solution containing CdSe QDs (square). The absorbance spectra of the pristine CdS and CdSe QDs in aqueous solutions used for the NRAs treatment are shown in inset.

Figure 4 shows diffuse reflectance absorption spectra of ZnO NRAs prior and after decoration with CdS and CdSe QDs. The absorption in Kubelka-Munk units of the different ZnO electrodes sensitized with QDs has been extracted from their diffuse reflectance using the relation: *F*(*R*) = (1*-R*)^2^/2*R*, where *R *is the measured diffuse reflectance. This presentation allows a direct comparison of the amount of QDs adsorbed on each sample. The intrinsic absorption of ZnO nanorods can be seen as a steep increase below 400 nm. The QD decorated ZnO NRAs show increased absorption from 400 to 450 nm for CdS and from 400 to 520 nm for CdSe, respectively, with additional features around 425 and 480 nm due to the size-dependent electronic transitions of QDs. These spectral features closely match the absorption maxima of aqueous colloidal solutions of CdS and CdSe QDs, inset in Figure 4, with a slight red-shift which is likely caused by the close packing of QDs deposited on ZnO [26]. From the data of Figure 4, we estimate approximately the same amount of CdS and CdSe QDs adsorbed on ZnO NRAs, which is also expected owing to their same surface ligands and a similar concentration of nanoparticles in solution. The absorption features of the both QDs materials are present for samples sequentially immersed in CdS and in CdSe QD solutions. Remarkably, the *in situ *ZnO NRA growth in a bath solution containing CdSe QDs results in a strong absorption enhancement with respect to the NRAs decorated with QDs.

**Figure 5:**
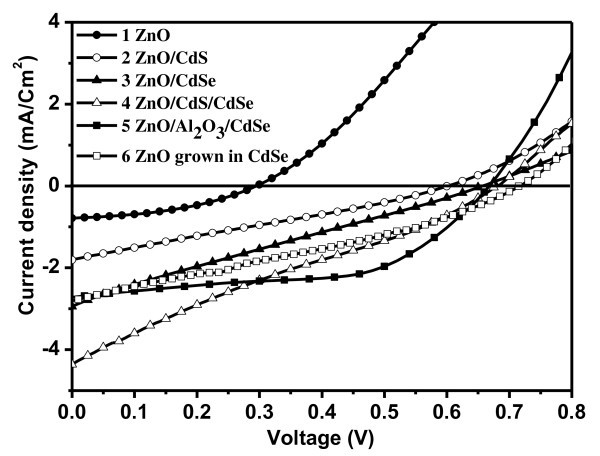
***J-V *characteristics of different QDSC**. ZnO nanorods (curve 1: filled circle), ZnO nanorods decorated with CdS QDs (curve 2: open circle), ZnO nanorods decorated with CdSe QDs (curve 3: filled triangle), ZnO nanorods decorated with both CdS and CdSe QDs (curve 4: open triangle), ZnO nanorods coated with Al_2_O_3 _and then decorated with CdSe QDs (curve 5: filled square), and ZnO nanorods *in situ *grown in a solution containing CdSe QDs (curve 6: open square).

The photovoltaic performance of QDSCs based on the vertically aligned ZnO NRAs fabricated on Zn substrates and decorated with CdS and CdSe QDs has been evaluated in a photoelectrochemical cell configuration with a liquid electrolyte containing the I_3_^-^/I^- ^redox couple with illumination through the Pt-coated FTO glass. The current density-voltage (*J*-*V*) characteristics for solar cells assembled from ZnO NRAs with and without QDs, which represent the performance averaged over 3-5 sample preparations are shown in Figure 5. For the non-sensitized ZnO NRAs based solar cells (curve 1 in Figure 5), the open-circuit voltage (*V*_OC_) and short circuit current (*J*_SC_) are low. The solar cells decorated with CdSe QDs (curve 3) perform better than those with CdS QDs (curve 2) in terms of the open-circuit voltage and the short-circuit current density, resulting in higher power conversion efficiency (0.46% for CdSe vs. 0.29% for CdS), which is attributed to the broader light absorption of CdSe compared with that of CdS (Figure 4). On the other hand, the FF of CdSe QDs based solar cells is smaller than that of CdS based solar cells, which can be related to the different electron recombination mechanism for the ZnO/CdS and ZnO/CdSe electrodes [27]. At the same time, as CdS solar cells present lower photocurrents, the voltage drop in the series resistance is lower for this type of solar cells, which also enhance the FF. The best performance has been achieved for ZnO nanorods simultaneously decorated with both CdS and CdSe QDs (curve 4), with *V*_OC _of 0.68 V and *J*_SC _of 4.36 mA/cm^2^. This shows an advantage of synergetic employment of different types of QDs as it has been discussed recently [12,28]. The performance of the CdSe QD decorated solar cell could be further improved by introducing an ultrathin (~2 nm) layer of Al_2_O_3 _grown by ALD on the surface of ZnO nanorods prior to their decoration with QDs. The deposition of Al_2_O_3 _shell resulted in a slight increase in *V*_OC _and decrease in *J*_SC _(curve 5 in Figure 5). However, the FF significantly improved from 0.24 to 0.55, leading to about 50% increase in power conversion efficiency from 0.46 to 0.99%. The increase in *V*_OC _has been attributed to the reduction of the electron recombination at the semiconductor/electrolyte interface with the passivation of the recombination sites at the ZnO surface by the Al_2_O_3 _coating, while the decrease in *J*_SC _resulted from partial inhibition of the electron injection at the semiconductor/QD interfaces [4,29]. Besides, the charge transfer at the Zn/electrolyte interface has been retarded resulting in a platform of the *J*-*V *curve for the lower potentials. Accordingly, an intimate contact between the ZnO NRAs and the CdSe QDs (as for the ZnO NRAs *in situ *grown in the presence of CdSe QDs which are presented in Figure 3c,d) could lead to an increase in *V*_OC _and FF due to the eliminated recombination sites at the ZnO/CdSe interface. Indeed this is observed in Figure 5 (curve 6), where *V*_OC _has further increased to 0.72 V without significant change in *J*_SC _(~2.81 mA/cm^2^). The performance characteristics of the solar cells illustrated in Figure 5 are summarized in Table 1.

**Table 1:** Photovoltaic performance of QDSC made of the vertically aligned ZnO nanorods fabricated on Zn substrates and decorated with CdS and CdSe QDsM

Electrode	***V***_**OC **_**(V)**	***J***_**SC **_**(mA/cm**^**2**^**)**	FF	*η *(%)
ZnO	0.28	0.78	0.41	0.09

ZnO/CdS	0.60	1.81	0.27	0.29

ZnO/CdSe	0.65	2.94	0.24	0.46

ZnO/(CdS and CdSe)	0.68	4.36	0.24	0.72

ZnO/Al_2_O_3_/CdSe	0.66	2.72	0.55	0.99

ZnO/CdSe (*in situ *grown)	0.72	2.81	0.30	0.62

## Conclusion

In summary, ZnO nanorods have been grown on Zn substrate by a simple one-step solution-based approach allowing for large-scale, low cost fabrication of vertically aligned arrays. The decoration of ZnO nanorods with CdS and CdSe QDs has been achieved by using aqueous-based QDs capped with a short ligand thioglycolic acid serving as molecular linker to ZnO nanorod surface. The photovoltaic performance of ZnO nanorods on Zn foil decorated with CdS and CdSe QDs has been evaluated in a photoelectrochemical solar cell configuration with a liquid triiodide/iodide electrolyte. The simultaneous use of CdS and CdSe QDs results in a higher open circuit voltage and short circuit current for co-sensitized structure in spite of a low FF. Power conversion efficiencies of approximately 1% were achieved using ZnO/Al_2_O_3_/CdSe electrode with an improved FF of 0.55. We have shown that simultaneous growth of the ZnO NRAs in the presence of CdSe QDs is possible and results in the improved *V*_OC _without compromising *J*_SC _and FF. Further work is on the way to develop devices with stable performance using, for example, TiO_2 _amorphous coating encapsulating the QDs [30], or QDs layer deposited on ZnO followed by a layer of Ruthenium dye. The last approach would benefit from the already mentioned advantage of improved light harvesting and charge extraction [31,32], and at the same time alleviate the problem of photocorrosion for QDs in contact with I_3_^-^/I^- ^redox couple and the problem of the instability of ZnO in contact with acidic dye [32].

## Abbreviations

ALD: atomic layer deposition; FFT: fast Fourier transform; FF: fill factor; HRTEM: high-resolution transmission electron microscope; NRAs: nanorod arrays; QDs: quantum dots; QDSCs: quantum dot sensitized solar cells; SEM: scanning electron microscopy; TEM: transmission electron microscope; TGA: thioglycolic acid; XRD: X-ray diffraction.

## Competing interests

The authors declare that they have no competing interests.

## Authors' contributions

CL carried out the preparation of ZnO samples and solar cell devices and drafted the manuscript. AV and AS prepared QDs and participated in absorption spectra measurements and in the preparation of the manuscript. XC and WZ carried out TEM and HRTEM characterization. HW, JX and CSL participated in the current density-voltage performances measurement and analysis. XX, AR and JZ participated in the revision of the manuscript and finalized the manuscript. All authors read and approved the final manuscript.
